# Prof. Dalton

**Published:** 1853-12

**Authors:** 


*Prof. Dalton.*—We take pleasure in informing our readers, through the following extract from the New York Medical Times, of the whereabouts and present employment of the gentleman who last winter occupied the tripod of this Journal, during the absence of our senior.

Dr. Dalton will soon commence his lectures and vivisections at the Buffalo Medical College, as usual.

"We observe that a change has been made in the arrangements for lectures at the College of Physicians and Surgeons, since the publication of the programme for the season.

Dr. Bartlett being prevented by illness from delivering his accustomed course, the subjects usually treated by him have been distributed among the other professors. In consequence of the extra duty thus devolving upon Dr. Clark, the Professor of Physiology and Pathology, a special series of lectures on the physiology of digestion and the nervous system is the course of delivery by *Dr. J. C. Dalton, jun.*, of the Preparatory School of this city.

Dr. Dalton has been for some time favorably known as an experimental physiologist, and his course at the Crosby St. School is liberally illustrated by *anæsthetic* vivisections—a humane and *American* refinement, by the way, which we recommended some time since, and which we are glad to see introduced. It is well known that, by means of gastric, biliary, and pancreatic fistulæ, established upon dogs and other animals, and by the analyses of the different salivary fluids, and the detection of sugar in the liver, most of the vexed questions as to the proper *role* of the various glands and other appendages attached to the alimentary canal, have been definitively settled within the last four or five years. So that there is no exaggeration in saying that the student, who is attentive to these lectures and experiments, may follow with his eyes, in most cases, all the successive changes to which the alimentary bolus is subjected, from the time it is first moistened by the buccal secretions till it has been prepared for absorption by the veins and lacteals.

We notice that, in addition to the experiments of Bernard and other physiologists, with which we have been made acquainted by recent journals, Dr Dalton has performed several, which we believe to have been designed by himself. His style of operating is rapid and brilliant; and, from the care with which he arranges the little but essential preliminaries and details, he is almost invariably successful.

Prof. Clark, thus relieved of a part of his course on physiology and pathology, takes a portion of the course on theory and practice, and thus enables Prof. Smith to supply the necessary instruction on materia medica, which the continued ill-health of Prof. Bartlett prevents him from resuming this season.

